# Metabolomic profiles of depression in Parkinson’s disease patients

**DOI:** 10.1038/s41531-025-01226-2

**Published:** 2025-12-11

**Authors:** Yuyuan Lin, Kimberly C. Paul, Dean P. Jones, Douglas I. Walker, Aline Duarte Folle, Irish Del Rosario, Yu Yu, Keren Zhang, Adrienne M. Keener, Jeff Bronstein, Beate Ritz

**Affiliations:** 1 Department of Epidemiology, UCLA Fielding School of Public Health, Los Angeles, CA USA; 2 Department of Neurology, UCLA David Geffen School of medicine, Los Angeles, CA USA; 3 Division of Pulmonary, Allergy, Critical Care and Sleep Medicine, Department of Medicine, Emory University School of Medicine, Atlanta, GA USA; 4 Department of Biochemistry, Emory University School of Medicine, Atlanta, GA USA; 5 Gangarosa Department of Environmental Health, Emory University Rollins School of Public Health, Atlanta, GA USA; 6 Center for Health Policy Research, UCLA Fielding School of Public Health, Los Angeles, CA USA; 7 Parkinson’s Disease Research, Education, and Clinical Center, Greater Los Angeles Veterans Affairs Medical Center, Los Angeles, CA USA

**Keywords:** Biomarkers, Diseases, Neurology, Neuroscience

## Abstract

Depression is a common non-motor symptom of Parkinson’s disease (PD), with poorly understood mechanisms. To explore whether there are dysregulated metabolic pathways among PD patients with depression, we analyzed serum samples of PD patients from a population-based case-control study (total *n* = 635) and performed metabolome-wide association and pathway analyses of depression in PD. We identified 212 metabolomic features associated with having ever received a depression diagnosis before PD and 213 features with higher Geriatric Depression Scale (GDS) scores (129 were annotated). Metabolic features we identified belonged to 14 pathways: glycerophospholipid metabolism for both outcomes and tryptophan, tyrosine, folate, biopterin, and sialic acid metabolism for those with higher GDS scores. An association with 6-hydroxy-1H-indole-3acetamide we observed likely indicates recent antidepressant treatment. These findings suggest that dysregulation in lipid and amino acid pathways, including tryptophan and tyrosine metabolism involved in neurotransmitter synthesis, may reflect altered neurochemical signaling and systemic metabolic changes related to depression in PD.

## Introduction

Parkinson’s disease (PD) is the second most common neurodegenerative disorder characterized by its key motor symptoms of tremor, rigidity, and bradykinesia. It is now, however, increasingly recognized that PD non-motor symptoms pose great challenges to the quality of life for PD patients and their caregivers. Depression, manifesting as persistent sadness, loss of interest in previously enjoyed activities, fatigue, and changes in sleep patterns, is one of the most common neuropsychiatric disturbances among PD patients. It is estimated that 50% of PD patients experience some form of depressive symptoms before or during the course of PD, and 35% of PD patients suffer from clinically significant depression^1^. Despite its high prevalence, depression in PD patients frequently remains undiagnosed and undertreated^2,3^. The pathophysiological underpinnings of this condition are still poorly understood. Some studies suggest that PD-related depression is associated with PD disease duration and features of progression, such as dosage of dopaminergic medication, severity of motor symptoms and cognitive decline^2,4^. But depression does not necessarily parallel the progression of physical symptoms^5^ and it is well-known that depression can precede PD by decades^6,7^. Hence, depression in PD patients may not simply result from PD pathology or be reactive to PD-associated disability, but contribute to a specific PD endophenotype possibly related not only to the degeneration or loss of innervation of cortical and subcortical dopaminergic but also noradrenergic and possibly serotoninergic systems^8,9^.

Metabolomics analyses offer distinct advantages in understanding disease mechanisms as they provide information on both changes in metabolism related to disease and metabolic responses to exogenous risk factors^10–12^. This approach has been applied to explore the pathogenesis of PD^13–16^, and an even larger number of studies explored the metabolome of major depression^17–19^. But only a few small studies have focused on PD-related depression^20^. For example, a targeted metabolomics study of 82 PD patients found plasma serotonin levels and its metabolite 5-hydroxyindoleacetic acid to decrease with depression score severity^21^, while another study of 63 PD patients (23 with depression) identified lipid and glucose metabolic pathways as being disturbed among those with depression^22^.

**Fig. 1:**
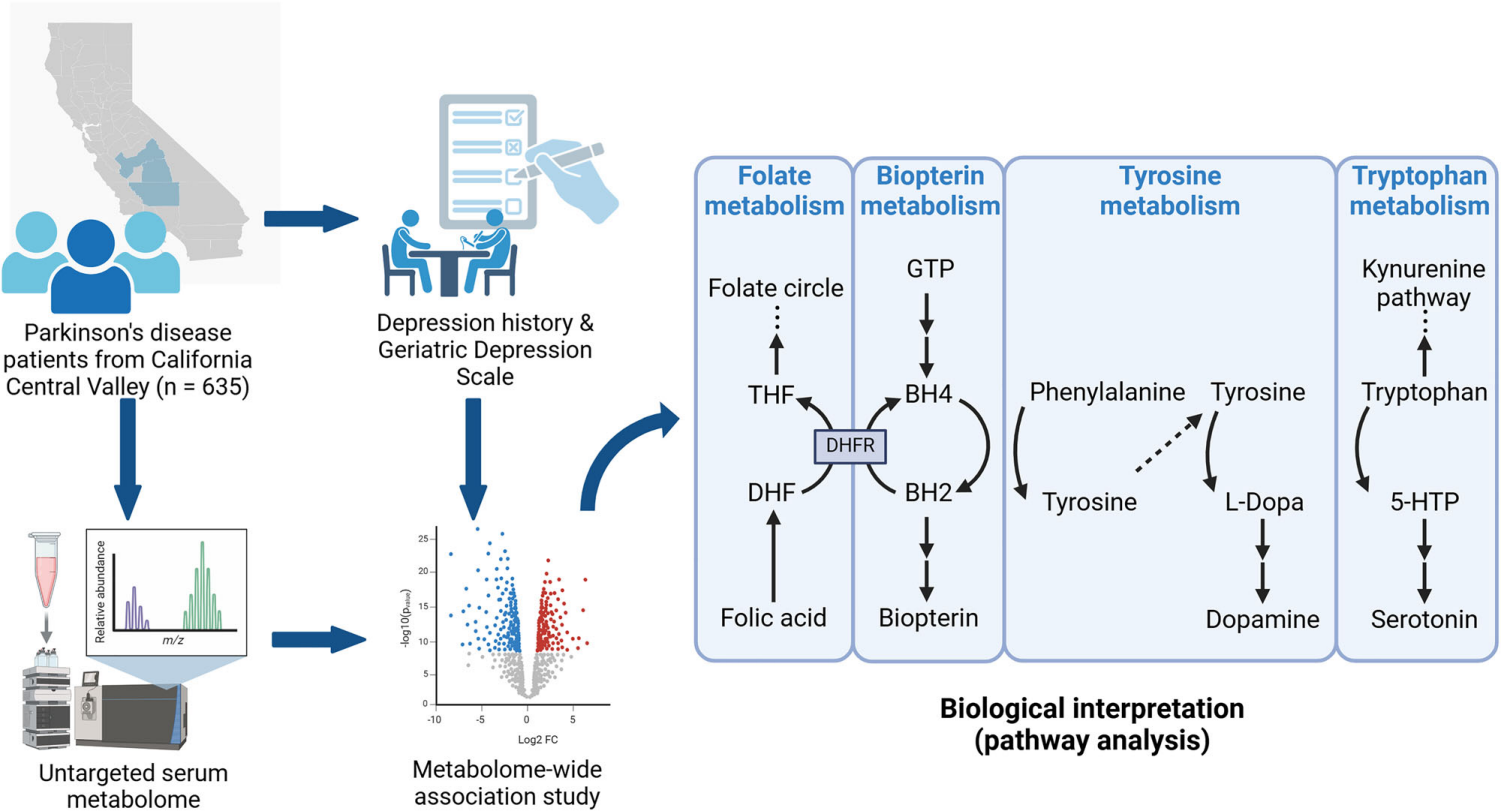
Summary of the study design and selected findings in dysregulated pathways. Abbreviations: THF, tetrahydrofolate; DHF, dihydrofolate; DHFR, dihydrofolate reductase; GTP, guanosine triphosphate; BH4, tetrahydrobiopterin; BH2, dihydrobiopterin; 5-HTP, 5-hydroxytryptophan. Created in BioRender. Lin, Y. (2025) https://BioRender.com/p06e449.

Based on existing evidence, we hypothesized that metabolic alterations occur in PD patients with depression. To investigate this, we performed an exploratory untargeted metabolomics analysis using serum samples from a large population-based study of PD, to identify metabolite features and pathways associated with depression among PD patients (Fig. 1). To our knowledge, our study is one of the first among population rather than clinic-based studies that apply untargeted metabolomics to research this topic.

## Results

### Study population

The study population included 635 PD patients from the UCLA Parkinson’s Environment and Gene (PEG) Study, of whom 158 (25%) had a depression diagnosis prior to their PD diagnosis. Patients with a history of depression had a higher baseline Geriatric Depression Scale (GDS) score (mean = 5.4, standard deviation [SD] = 3.9) compared to those without depression (mean = 3.0, SD = 2.8). They also had a higher prevalence of smoking history, were slightly younger on average, and included a greater proportion of females. Patterns of education, PD duration, and race/ethnicity were similar between patients with and without depression. Detailed demographic information is presented in Table 1.

**Table 1:** Demographic information for the study population

Variable^a^	Overall, *N* = 635	Depression before PD diagnosis	
		Yes, *N* = 158	No, *N* = 477
**Baseline GDS score**	3.6 (3.3)	5.4 (3.9)	3.0 (2.8)
**Age at PD diagnosis**	66.9 (10.3)	65.2 (9.8)	67.4 (10.4)
**Years in school**	13.7 (4.6)	13.5 (4.8)	13.8 (4.5)
**PD duration**	3.0 (2.6)	3.0 (2.5)	3.0 (2.6)
**PEG study waives**			
PEG 1 (2001–2007)	281 (44%)	75 (47%)	206 (43%)
PEG 2 (2011–2018)	354 (56%)	83 (53%)	271 (57%)
**Sex**			
Male	405 (64%)	94 (59%)	311 (65%)
Female	230 (36%)	64 (41%)	166 (35%)
**Race and ethnicity**			
Non-Hispanic white	478 (75%)	124 (78%)	354 (74%)
Others	157 (25%)	34 (22%)	123 (26%)
**Smoking history**			
Never smoke	339 (53%)	73 (46%)	266 (56%)
Ever smoke	296 (47%)	85 (54%)	211 (44%)

^a^Variable distributions are reported as mean (SD) for continuous variables and *n* (%) for categorical variables.

### Metabolome-wide association analysis results

**Fig. 2:**
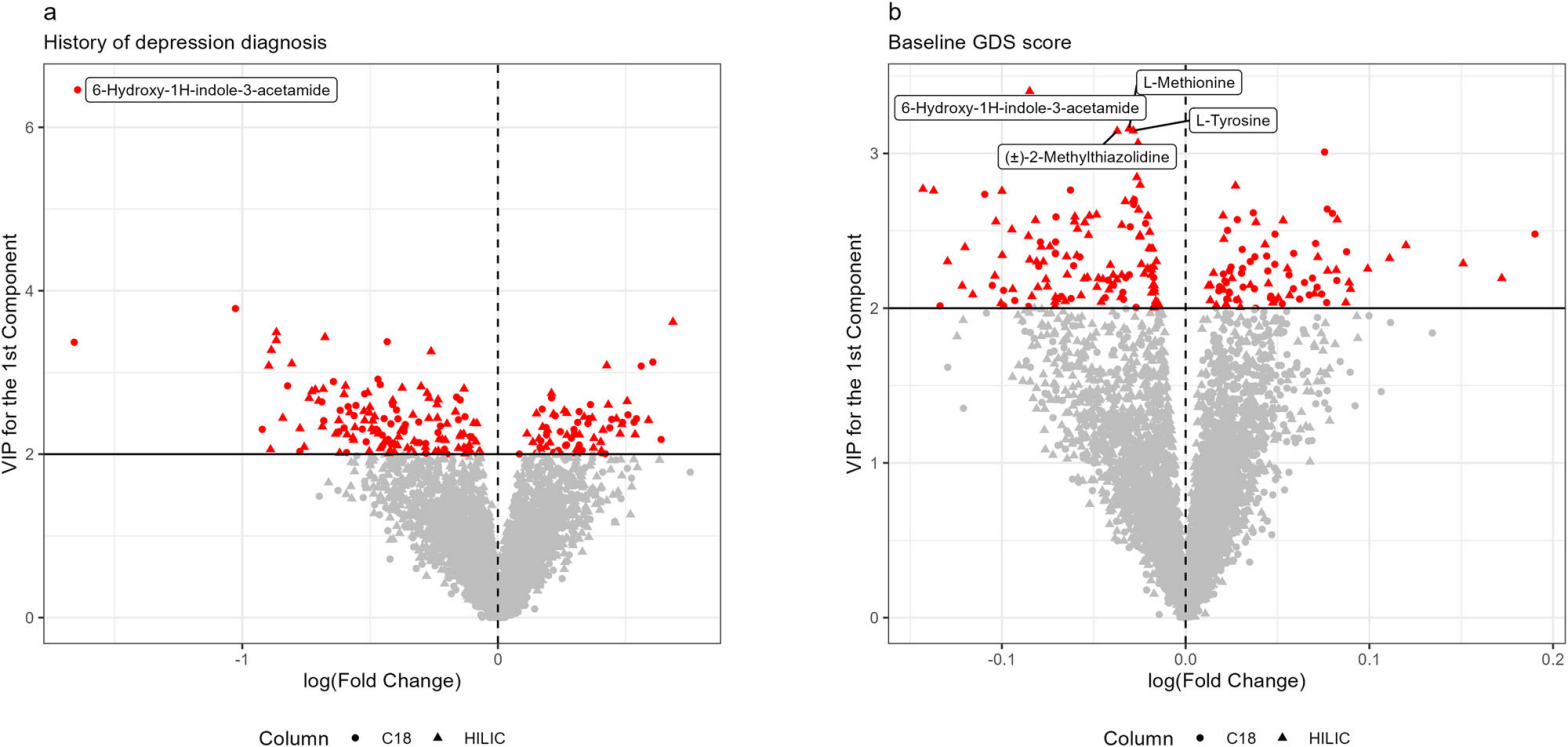
Volcano plots showing the MWAS results. The Y-axis represents the VIP values for the 1st component from PLS(-DA) analyses, and the X-axis is the log-transformed fold change from empirical Bayesian analyses. **a**, **b** Show the results for Analysis 1 (history of depression diagnosis) and 2 (baseline GDS score), respectively. Peaks that are significant in regression-based MWAS or empirical Bayesian models are named in the figures.

For untargeted high-resolution metabolomics (HRM), we profiled serum samples from the PD patients using two chromatography columns: including hydrophilic interaction (HILIC) chromatography with positive electrospray ionization (ESI) and C18 chromatography with negative ESI. After preprocessing and quality control steps, 4762 metabolomic features (2,046 from the C18 column and 2716 from the HILIC column) were detected and included in the subsequent analysis. For the metabolome-wide association study (MWAS), we employed three analytical approaches—partial least squares (PLS) and partial least squares discriminant analysis (PLS-DA), empirical Bayes models, and regression models—using two depression outcomes: binary depression history and continuous baseline GDS score. In the first analysis using binary depression history as the outcome, the PLS-DA model identified 90 features from the C18 column and 122 features from the HILIC column with variable importance in projection (VIP) > 2 after adjusting for potential confounders (sex, age, race/ethnicity, education level, smoking status, PD duration, and PEG study wave) (Fig. 2a). In the second analysis using a continuous GDS score as the outcome, the PLS analysis identified 81 and 132 features from C18 and HILIC columns respectively with VIP > 2 (Fig. 2b). The empirical Bayes and regression models picked identical features for both analyses. One feature from the C18 column was associated with depression history and 5 features from the HILIC column were associated with increased baseline GDS score after multiplicity correction. The full results of MWAS are shown in Supplementary Data 1 and 2.

Of the depression-associated metabolomic features, we successfully annotated 129 (50 from the C18 column and 79 from the HILIC column) to known metabolites in chemical databases using either the xMSannotator or an in-house library. Among these, 52 were mapped to single compounds, and the rest (77) have multiple annotations. In the rest of the paper, we use “annotated metabolites” to refer to features that were successfully matched to compound databases, and “associated features” to denote those that reached statistical significance in the MWAS. Among annotated metabolites, amino acids, lipids, and lipid-like molecules are leading categories (Supplementary Fig. 2). The top metabolite in the PLS-DA models from both Analysis 1 and Analysis 2 was **6-Hydroxy-1H-indole-3-acetamide** (m/z = 189.067, RT = 63.4 s, from C18 Column), with a VIP score of 6.46 in Analysis 1 and 3.24 in Analysis 2. It is also the only feature identified by the empirical Bayes and the regression model with an false discovery rate (FDR) < 0.05 in Analysis 1. In Analysis 2, 3 features from HILIC column were identified by empirical Bayes and regression models, including **L-Methionine** (m/z = 194.022, RT = 65.1 s), **L-Tyrosine** (m/z = 182.081, RT = 72.2 s), and **(±)-2-Methylthiazolidine** (m/z = 104.053, RT = 65.9 s). In general, the two analyses using different depression outcome variables identified different sets of metabolomic features with some overlap in linked features. Supplementary Fig. 3 shows the correlation between regression coefficients from the two analyses and the overlap of identified metabolites. Pearson’s correlation coefficients between PLS loadings from the two analyses were 0.436 for the C18 column and 0.395 for the HILIC column, indicating moderate correlations between the results for the two depression outcome measures.

### Enriched pathway analysis

**Fig. 3:**
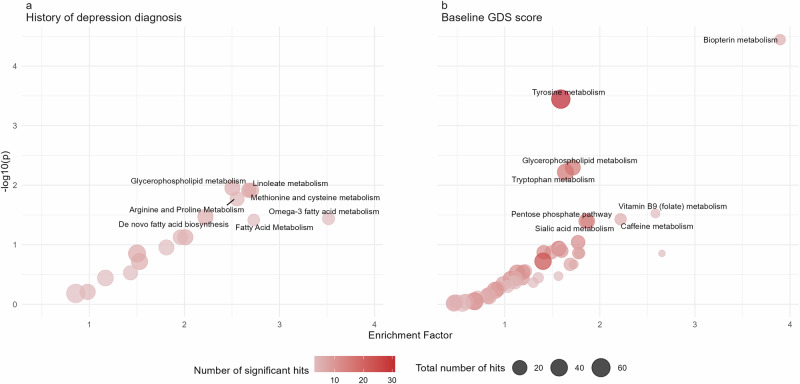
Pathway analyses results using “Mummichog”. **a** Shows the results from the 1st analysis (using binary depression diagnosis as the outcome); **b** shows the results from the 2nd analysis (using baseline GDS score as the outcome). The enrichment factor is the ratio between the number of significant peaks and the expected number of peaks within that pathway. Pathways with 3 or more peaks associated with the outcome are included in the figures. The position of bubbles was jittered to avoid overlapping.

Using the Mummichog method, we examined whether specific metabolic pathways were overrepresented among the associated metabolomic features. From Analysis 1 (history of clinical depression), 7 pathways were overrepresented at a *p* < 0.05 and contained at least 3 significant metabolomic features and 8 pathways were identified in Analysis 2 (GDS). All overrepresented pathways are shown in Table 2. Glycerophospholipid metabolism was selected in both analyses. Most of the overrepresented pathways are involved in lipid and amino acid metabolism, which are essential for cellular function. Additionally, identified pathways encompass biopterin metabolism, folate metabolism, sialic acid metabolism, caffeine metabolism, and the pentose phosphate pathway (Fig. 3).

**Table 2:** List of enriched pathways using “Mummichog” method in the main analysis

	Pathway size	Total Number of peaks	Number of significant peaks	*P* value
**Analysis 1 (binary depression history)**				
Glycerophospholipid metabolism	156	25	6	0.011
Linoleate metabolism	46	19	9	0.012
Methionine and Cysteine metabolism	94	21	6	0.012
Arginine and Proline Metabolism	45	19	4	0.017
De novo fatty acid biosynthesis	106	26	6	0.034
Omega-3 fatty acid metabolism	39	10	3	0.036
Fatty acid metabolism	63	9	3	0.039
**Analysis 2 (baseline GDS score)**				
Biopterin metabolism	22	6	6	<0.001
Tyrosine metabolism	160	65	31	<0.001
Glycerophospholipid metabolism	156	25	17	0.005
Tryptophan metabolism	94	34	17	0.006
Vitamin B9 (folate) metabolism	33	4	3	0.030
Caffeine metabolism	11	8	6	0.037
Sialic acid metabolism	107	18	13	0.040
Pentose phosphate pathway	37	21	13	0.040

### Supplementary analysis results

In sensitivity analyses, we first additionally adjusted for PD-related clinical covariates measured at baseline, including levodopa-equivalent daily dosage (LEDD), the Unified Parkinson’s Disease Rating Scale Part III (UPDRS-III) score, the Mini Mental State Examination (MMSE) score, and an indicator for probable presence of rapid eye movement sleep behavior disorder (RBD) features^23^. The MWAS results compared to the main analysis are presented in Supplementary Data 3–6. In summary, after adjusting for LEDD, 66 annotated metabolomic features with VIP > 2 were associated with depression history, and 65 features with baseline GDS. After adjusting for baseline UPDRS-III, MMSE, and the RBD indicator, 72 annotated features were associated with depression history and 59 with baseline GDS. Most features overlap with those from the main analyses, and PLS loadings were highly correlated for all analyses (Pearson’s correlation > 0.9, Supplementary Figs. 4 and 5). In the regression and empirical Bayes models, the FDR for 6-Hydroxy-1H-indole-3-acetamide remained significant after adjustment for these clinical features. However, the other three metabolites, while still top of the list, failed to reach formal statistical significance. The pathway analysis after adjustment still selected similar pathways. Adjusting for LEDD, Mummichog pathway analysis identified an almost identical set of pathways as our main analysis (Supplementary Table 1). When further adjusting for UPDRS-III, MMSE, and the RBD indicator, a few additional pathways emerged, while the previously significant pathways largely remained among the top-ranked results (Supplementary Table 2). Thus, the adjustment for other PD-related clinical covariates did not change the main results of the study overall.

Second, we performed a sensitivity analysis adjusting for Hoehn and Yahr (H&Y) stage, followed by a stratified analysis of early (H&Y ≤ 2) and late (H&Y ≥ 2.5) PD stages. With additional adjustment for H&Y stage, the associated metabolomic features largely overlapped with those identified in the main analysis (Supplementary Data 7 and 8). The correlations of PLS loadings were 0.98 for the depression history analysis and 0.96 for the baseline GDS score analysis (Supplementary Fig. 6). Mummichog pathway analysis also identified the same set of pathways associated with baseline GDS score. For depression history, fatty acid–related pathways were selected as in the main analysis, whereas glycerophospholipid metabolism, linoleate metabolism, and amino acids–related pathways did not reach statistical significance (Supplementary Table 3). In the stratified analysis, 6-Hydroxy-1H-indole-3-acetamide was the only metabolite that reached statistical significance in both the regression and empirical Bayes models, and this association was only observed among patients who were still in an early H&Y stage. The PLS and PLS-DA models identified a comparable number of features (~200 with VIP > 2) across all analyses. The lists of annotated features with VIP > 2 are provided in Supplementary Data 9 and 10.

**Fig. 4:**
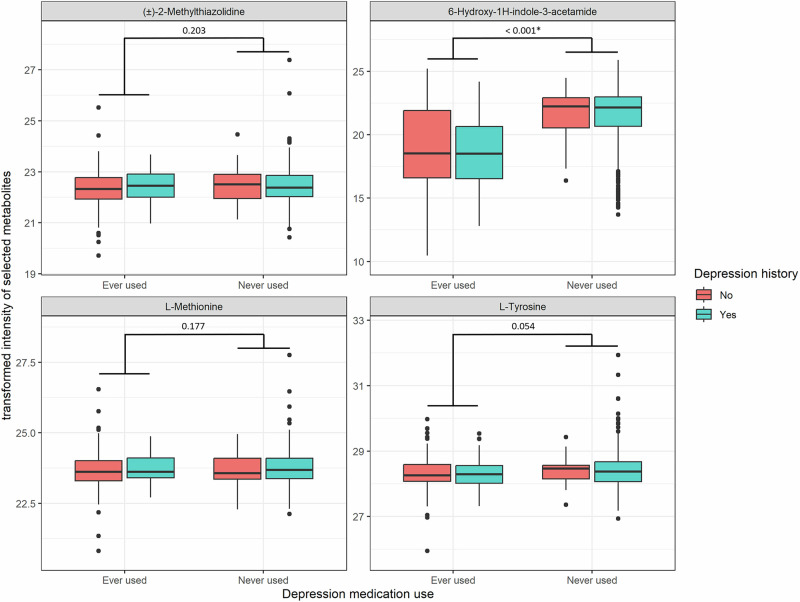
The boxplot of transformed intensity of MWAS-selected metabolites stratified by depression diagnosis and depression medication use history. The *p* values were obtained from two-sample *t*-tests comparing individuals who had ever used antidepressants with those who had never used them. The boxplot follows Tukey’s style, displaying the median, the 1st and 3rd quartiles (hinges), and whiskers extending to 1.5 times the interquartile range from the hinges.

Considering medication could influence the metabolomic profile of patients with depression, we further conducted an exploratory analysis for the top 4 metabolites (6-Hydroxy-1H-indole-3-acetamide, L-Methionine, L-Tyrosine, and (±)-2-Methylthiazolidine) to see whether the intensity was affected by antidepressant use. Among the 4 top metabolites, only 6-Hydroxy-1H-indole-3-acetamide was found to be associated with antidepressant use (Fig. 4). Specifically, the transformed intensity of 6-Hydroxy-1H-indole-3-acetamide was significantly lower in patients who used selective serotonin reuptake inhibitors (SSRI) or serotonin-norepinephrine reuptake inhibitors (SNRI) within 1 year compared to people who never used it (Supplementary Table 5).

We also assessed the associations between the top four metabolites we identified in PD patients with depression in control participants we have enrolled in the PEG study. The level of 6-hydroxy-1H-indole-3-acetamide was lower in PD controls with a history of depression compared to those without (regression coefficient = −0.16, *p* = 0.04), and also inversely associated with baseline GDS scores (regression coefficient = −0.19, *p* = 0.04). The remaining three metabolites (L-Methionine, L-Tyrosine, and (±)-2-Methylthiazolidine) did not show any association with depression in controls without PD (Supplementary Table 6).

## Discussion

Our study used HRM to investigate potential metabolic perturbations related to two depression outcomes in PD patients: depression history before PD onset and GDS score at baseline interview. Relying on serum samples from 635 PD patients of whom 158 had a history of depression, 69 annotated metabolites were found to be associated with a history of clinical depression before a PD diagnosis, and 73 annotated metabolites were associated with a higher GDS score at baseline examination. Among these metabolites, four were also identified by empirical Bayes and regression models. We found evidence for dysregulation of 14 metabolic pathways through overrepresentation analysis, including multiple lipid and amino acid metabolism pathways. The correlation between the two outcome analyses was moderate even though they showed, as one would expect, some overlap. The discrepancies most likely mean that these measures capture different aspects of depression. Depression history reflects depressive traits and possible episodes that occurred prior to PD onset, whereas GDS scores reflect current depressive symptoms at the time of interview when we also collected blood samples used for metabolomics. These complementary measures allowed for a more comprehensive assessment of metabolic changes in terms of traits and chronic states versus an acute state in which PD patients showed symptoms of depression.

Only one previous epidemiologic study used an untargeted metabolomics approach to study depression among PD patients and identify 85 differentially abundant metabolites that distinguished PD patients with and without depression^22^. In this study, the majority of the associated metabolites were lipids and lipid-like molecules, with both lipid and glucose metabolism altered among the patients with depression^22^. Similarly, lipid and lipid-like molecules also represent one of the leading categories of depression associated metabolites in our study with glycerophospholipid metabolism being among the top pathways identified in our study. We have previously found that glycerophospholipids—key components of cell membranes—are dysregulated in PD patients compared to controls^15^, as also reported for PD patients in other studies^24^, and also found in animals models^25,26^. A systematic review of clinical and animal metabolomics research also indicated that glycerophospholipid metabolism was one of the disturbed pathways in both types of studies^18^, and a large pooled MWAS of major depression further supported lipid dysregulation as a key mechanism^17^. Our findings on lipid pathways are consistent with these studies and suggest even more extensive dysregulation of lipid metabolism pathways among PD patients affected by depression.

Among all metabolites identified in the current study, 6-hydroxy-1H-indole-3-acetamide showed the strongest association with depression, as lower levels of this metabolite were observed in both PD patients and controls with a history of clinical depression and among those with a higher GDS score at baseline. 6-Hydroxy-1H-indole-3-acetamide is a hydroxyindole. Although there is limited research on this specific metabolite, one previous metabolomics study also identified 6-Hydroxy-1H-indole-3-acetamide as being decreased in plasma samples from post-stroke depression patients^27^. The class of hydroxyindole compounds, particularly those involving serotonin, has a well-known involvement with depression^28^. Cerebrospinal fluid levels of 5-hydroxyindoleacetic acid (the major metabolite of serotonin) have been reported to be decreased in PD patients with depression compared to those without depression^29^. Interestingly, in our supplementary analysis, this metabolite was found to be strongly associated with current antidepressants use, especially SSRI and SNRI. This suggests that the metabolite is downregulated by treatment with serotonin reuptake inhibition and possibly is a biomarker for recent SSRI and SNRI use. Further study is required to better understand the role of this metabolite in the response to treatment of depression with these drug classes.

We additionally found that certain amino acids and related pathways were associated with depression in PD, especially in the analysis using the GDS score as the outcome. Among top metabolites identified in the study, tyrosine was observed to be less abundant among patients with a higher GDS score. In the pathway analysis, we found that tyrosine metabolism and tryptophan metabolism pathways are associated with severe depressive symptoms. As the precursor of the essential monoamine neurotransmitter serotonin, tryptophan and its metabolic pathways are closely involved in depression^30^. In PD, lower levels of tryptophan have previously been observed in the plasma of PD patients^31^. Additionally, abnormalities in the kynurenine pathway—the primary metabolic pathway of tryptophan—are linked to pathophysiological pathways important for both conditions, PD and depression. Higher levels of the neurotoxic to neuroprotective kynurenine metabolite ratio (quinolinic/kynurenic acid ratio) have been observed in both PD patients and patients with major depression^32,33^. Our study findings align with previous research, supporting the hypothesis that tryptophan metabolism, especially the kynurenine pathway, might play a key role in the mechanisms underlying the disease.

Tyrosine, on the other hand, is the precursor of the catecholamine series of neurotransmitters, and thus plays an important role in mood and motivational behavior regulation^34^. Reduced intensity of tyrosine in serum and dysregulation of its metabolism found in our study for PD patients with depression thus makes biologic sense. Notably, we found dysregulation in biopterin and folate metabolism among PD patients with more pronounced depressive symptoms. The fully reduced form of biopterin—tetrahydrobiopterin (BH4)—is a pivotal enzymatic cofactor for the degradation of phenylalanine to tyrosine, and the synthesis of serotonin and dopamine. Given the fundamental role of BH4 in neurotransmitter biosynthesis, a reduced level of BH4 has been observed in many neurological diseases including PD and depression^35^. Folate metabolism, linked to BH4 through the shared enzyme dihydrofolate reductase, is also thought to influence PD and depression risk via homocysteine metabolism^35,36^. Our study results are consistent with disease mechanisms that involve biopterin, folate, and aromatic amino acids essential for neurotransmitter synthesis. We also observe altered sialic acid metabolism in the same patient group, aligning with a prior study reporting reduced plasma sialic acid levels in patients with depression^37^. Interestingly, these dysregulations were found in patients with increased GDS scores but not in patients with a depression history, suggesting they are marking acute depression states.

The current study has several strengths. Our study is among the largest studies of depression in PD that applied a metabolomics approach. The relatively large sample size allowed us to explore the possible perturbations in metabolic profiles among PD patients with depression relative to patients without depression using untargeted metabolomics. We also assessed two different depression outcomes—a history of clinical depression prior to PD diagnosis and current depressive symptoms measured by the GDS at the baseline interview. Given the fact that depression is usually overlooked and underdiagnosed among PD patients, utilizing the GDS increased the chance to identify depressive symptoms independent of a clinical diagnosis. Several limitations, however, exist. First, the identification of unknown (not annotatable) metabolites remains a major challenge^13^. As is common in untargeted MWAS, we were only able to annotate a subset of the associated metabolomic features. Important metabolites may currently remain without annotation. Another limitation of the study is the timing of bio-sample collection and interview. Given that we obtained depressive symptom information and the serum sample at about the same time, we were unable to establish temporality between the metabolomic profile and current depressive symptoms measured by the GDS. The metabolic alterations we observed thus may represent different disease phases, such as perturbations contributing to depression as well as physiological reactions to symptom progression or treatment. Additionally, the history of a clinical depression diagnosis was self-reported. Depression in PD is commonly undiagnosed, raising the concern of nondifferential misclassification of the outcome. A limitation of the Mummichog approach used for pathway analysis is that it relies on putative metabolite annotation, which may introduce uncertainty and potential false positives in pathway results. Our untargeted approach is mainly explorative and hypothesis generating, and we hope that our results inspire further investigations into underlying molecular mechanisms contributing to depression in PD patients.

In summary, we utilized HRM techniques to identify altered metabolic profiles for PD patients with depression in a large community-based study population. Our study identified 129 metabolites as being associated with depression in PD relative to PD without depression. The metabolite strongly distinguishing these two groups, 6-Hydroxy-1H-indole-3-acetamide, may be related to antidepressant-treatment. Importantly, we also identified plausible metabolic pathways linked to depression in our patients that have also been previously linked to major depression or to PD in general, including tryptophan, tyrosine, biopterin, and glycerophospholipid metabolism, suggesting that dysregulation of these essential pathways may be a part of depression in PD.

## Methods

### Study population

Participants in the UCLA PEG Study, an ongoing population-based case-control study of PD conducted in agricultural regions of the California Central Valley, contributed to this study (for details see^23,38,39^). Briefly, PD patients were recruited among current residents from California’s Central Valley from 2001 to 2018 in two separate study waves. Case eligibility criteria include (1) having a PD diagnosis within 5 years of recruitment, (2) currently residing in one of the three research counties (Fresno, Kern, and Tulare) and having lived in California for at least 5 years before diagnosis, (3) being confirmed by UCLA movement disorder specialists as having “probable” or “possible” PD at a study exam^40^, (4) not having other diagnosed neurological conditions, serious psychiatric conditions, or being in the last stages of a terminal illness, and (5) consenting to participate the study. We collected demographic, lifestyle, and medical information via structured interviews and questionnaires from each participant. Participants were asked to provide blood samples at the baseline and during follow-up visits. In total, we screened 3881 potential participants, among whom 1279 were eligible and 862 patients were enrolled in the study. For this study, we included all PD patients who provided serum samples at baseline in either the first or second study wave (PEG1 from 2001 to 2007, PEG2 from 2011 to 2018). For 635 patients, serum samples were available at the time we conducted metabolomic analyses. The study recruitment information for each study wave is shown in Supplementary Fig. 1. The study was conducted in accordance with the Declaration of Helsinki and approved by the Institutional Review Boards of the University of California Los Angeles. All participants were informed of the procedures and their rights and were provided with written informed consent.

### High-resolution metabolomics profiling

Serum metabolomic features were measured by HRM profiling using liquid chromatography with mass spectrometry (LC-MS), as documented elsewhere^11,15^. Briefly, serum samples were collected, centrifuged, and aliquoted at time of the exam, and then stored at −80 °C before being sent to our collaborators at Emory University for profiling. The analytic method used was a dual-column, dual-polarity approach, including HILIC chromatography with positive ESI and C18 chromatography with negative ESI. The serum samples were analyzed in two analytical runs, with each run consisting of 15 batches. Quality control samples were included in each batch to evaluate system performance. Quality control and data preprocessing steps before statistical analysis are presented in Supplementary Fig. 1. Extracted samples were analyzed in triplicate. Metabolomic features were included in the steps described next if the average coefficient of variation among technical replicates was <30% and the Pearson correlation >0.9. To combine the data from the two analytical runs, feature alignment was conducted by adjusting the retention time using apLCMS in R^41^. Subsequently, metabolomic features detected in less than 50% of all samples were filtered out. Missing values in the metabolomic matrix were imputed using the feature-wise non-zero minimum value. After that, we median normalized the metabolomics matrix and then corrected for batch effects to get the final metabolomic feature matrix for analysis.

### Depressive outcomes and covariates

We collected basic demographic data, such as year of birth, sex, race, ethnicity, and educational level and a medical history at baseline, including a history of depression (ever having been given a diagnosis of depression and age of being diagnosed), antidepressant and PD medication use. We also evaluated current depressive symptoms using the 15-item Geriatric Depression Scale (GDS-15), a commonly used screening tool to identify depressive symptoms based on self-reporting. Previous research has validated and recommended the GDS as an appropriate tool to measure and screen for depression among PD patients^42,43^. We have also validated that GDS-15 is a successful screening tool for depression in PD among PEG1 patients, where we calculated 85% sensitivity and 79% specificity compared to a Structured Clinical Interview for the DSM-IV depression module (SCID) diagnosis of minor or major depression^44^. All questions in GDS-15 ask for a dichotomous response (yes/no) and are added up to generate a final score ranging between 0 and 15. A GDS score greater or equal to 5 suggests depression^42^. In the main analysis, we used the binary self-reported depression history before PD diagnosis (Analysis 1) and the continuous GDS-15 score at baseline (Analysis 2) as the depressive outcome.

The following covariates were included in statistical models as potential confounders: age at PD diagnosis (continuous), sex (binary, male or female), race and ethnicity (binary, non-Hispanic white or other race/ethnicity), smoking history (binary, never or ever smoke), educational level (continuous, years in school), PD duration at baseline (continuous, years), and PEG study wave (binary, PEG1 or PEG2).

### Metabolome-wide association analysis

To identify metabolites associated with depression history or an increasing GDS score at baseline in PD patients, we conducted an MWAS using three distinct methods: PLS/PLS-DA models, empirical Bayes models, and regression models. PLS and PLS-DA models are frequently used techniques in metabolomics analysis, which are constructed by maximizing the covariance between the independent variables (metabolomic features) and the outcome variables in high-dimension and finding a linear subspace of the predictors^45^. These models enable us to account for the correlation among metabolites. The PLS-DA model was fitted for binary depression history; the PLS model for the continuous GDS outcome. To adjust for potential confounders, we first regressed metabolomic features on the factors to be controlled and then used the residuals as the input matrix for PLS/PLS-DA models. Features with VIP scores greater than 2 were considered significant in the PLS/PLS-DA analysis. Empirical Bayes methods borrow information between features to stabilize estimates and improve statistical power^46^. Employing these models, we regressed the transformed intensity of metabolites on either the depression history or the baseline GDS score, adjusting for all potential confounders. Lastly, logistic models and multiple linear regression models were fitted respectively for binary depression history and continuous GDS score outcomes to estimate the strength of association between each metabolomic feature and depression, which were thereafter used as input for pathway analyses. We also report FDR to account for the multiplicity in regression models and empirical Bayes models.

### Pathway analysis and metabolites annotation

We carried out pathway analyses based on the results of univariate MWAS using the “Mummichog” approach included in “MetaboAnalystR” package (version 4.0)^47^. “Mummichog” is a frequently applied approach in metabolomics research, which uses a permutation-based framework that accounts for the complexity of untargeted mass spectral data^48^. The input matrix included column type (positive or negative), mass/charge ratio, retention time, estimated effect size (regression coefficient), and the *p* value for each metabolomic feature. The ion mode was set to mixed (including positive and negative columns at the same time) and the significance threshold was set to uncorrected *P* < 0.05. Enriched metabolic pathways were selected and interpreted if the pathway overrepresentation *p* value was less than 0.05 and the pathway contained 3 or more metabolites associated with depression in the MWAS.

For metabolite annotation, we first matched the metabolomic features identified in the MWAS to the metabolites from the in-house library, which is a database of detected and quantified metabolites established at Emory University^49,50^. In addition, we annotated the metabolomic features using the “xMSannotator” package^51^. The chemical databases used in xMSannotator include HMDB, KEGG, T3DB, and LipidMaps. The tolerance for the difference in retention time was set to less than 30 s and the difference in mass-to-charge ratio was set to 10 ppm. Some of the features were matched to multiple compounds. Annotations were included and interpreted only if the confidence level is greater than 0^51^. The “Mummichog” package also provides annotation for some metabolomic features, which was used as another validation for the annotated results.

### Supplementary analysis

We included LEDD as an extra covariate in the MWAS and compared results to models without LEDD to account for potential confounding by PD medication use. We further extended the analysis to control for additional baseline clinical factors, including motor symptom severity measured by the UPDRS-III, cognitive function assessed by the MMSE, and an indicator for probable presence of RBD features^23^, given these clinical characteristics are closely related with depression in PD. We applied the same analytic methods as in the main analysis.

Considering that metabolic profiles may vary across different stages of PD progression, we also controlled for H&Y stage and conducted a stratified analysis by disease stage (early stage: H&Y ≤ 2; late stage: H&Y ≥ 2.5) to examine whether the identified association were consistent across different severity levels of PD.

Additionally, given the possible impact of antidepressants on metabolism, we performed an exploratory analysis to examine how the use of different types of antidepressants influences the intensity of MWAS-picked metabolites. The antidepressants reported by patients were categorized as 5 types: SSRI, SNRI, atypical antidepressants, benzodiazepines, and others (Supplementary Table 4). The transformed intensity of selected metabolites was then regressed on the use history of each type of antidepressant according to time of use (currently using, i.e., within the past year, used more than 1 year ago, or never used), with and without adjusting for covariates.

Finally, we examined the top MWAS-associated peaks in the control group with available metabolomics data from the PEG study (*n* = 253) using linear and logistic regression models to evaluate whether these associations were specific to depression in PD. Models were adjusted for age at baseline interview, sex, education level, smoking history, and race/ethnicity as covariates.

## Supplementary information

Supplementary materials

Supplemental data

## Data Availability

The metabolomics data used in this study are publicly available on Metabolomics Workbench (Project ID: PR001964, 10.21228/M8VD96).
