# Supplementary material for: Metabolomic profiles of depression in Parkinson’s disease patients
Source: NPJ Parkinsons Dis. 2025 Dec 11;12:9. doi: 10.1038/s41531-025-01226-2 (PMC12789418; doi:10.1038/s41531-025-01226-2)
Supplement: Supplementary file 1 — Supplementary materials [file 41531_2025_1226_MOESM1_ESM.pdf]

Supplemental Figure 1

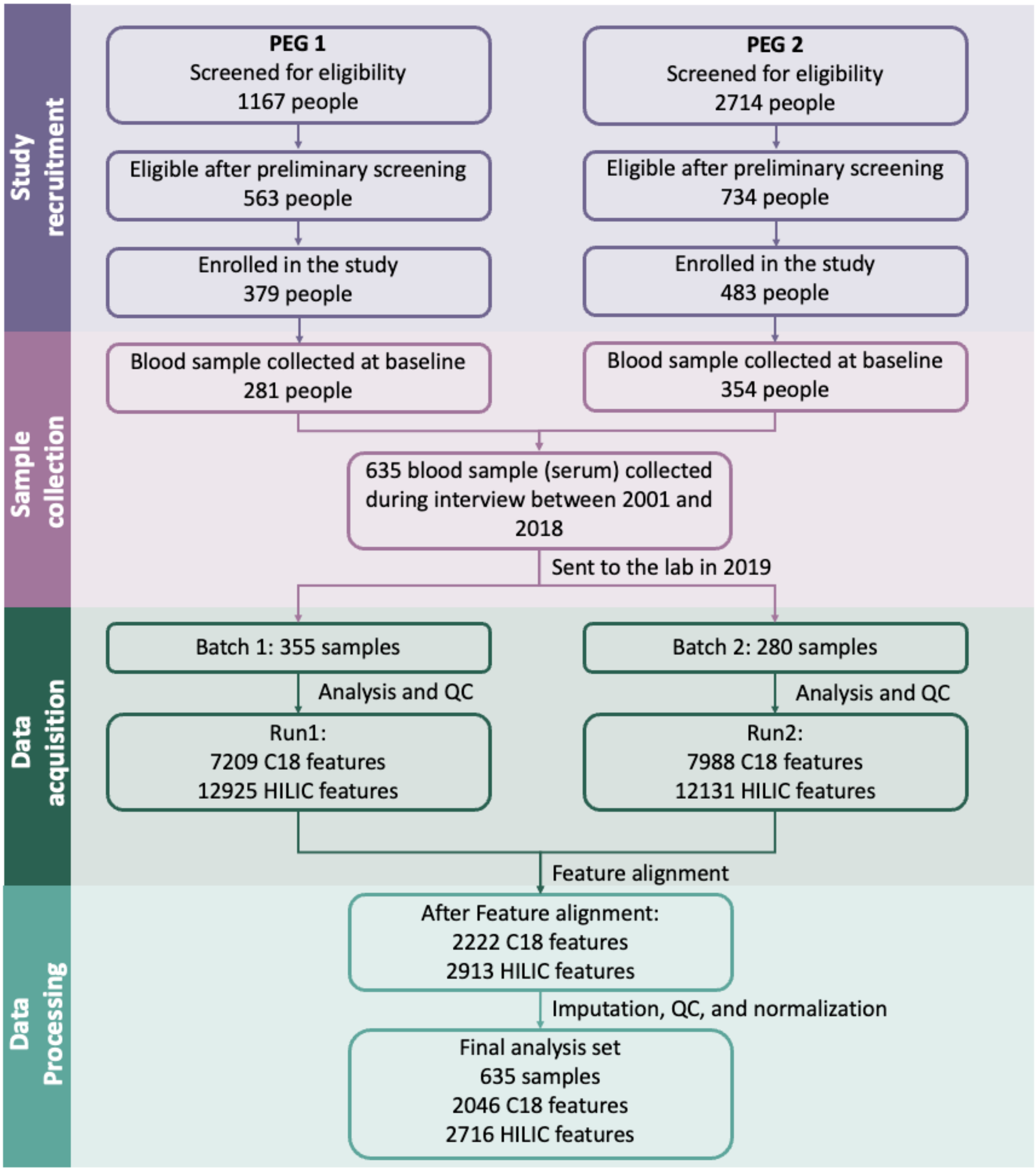

Fig. S1 Flowchart showing enrollment, data preprocessing, and quality control steps for the metabolomics analysis.

Supplemental Figure 2

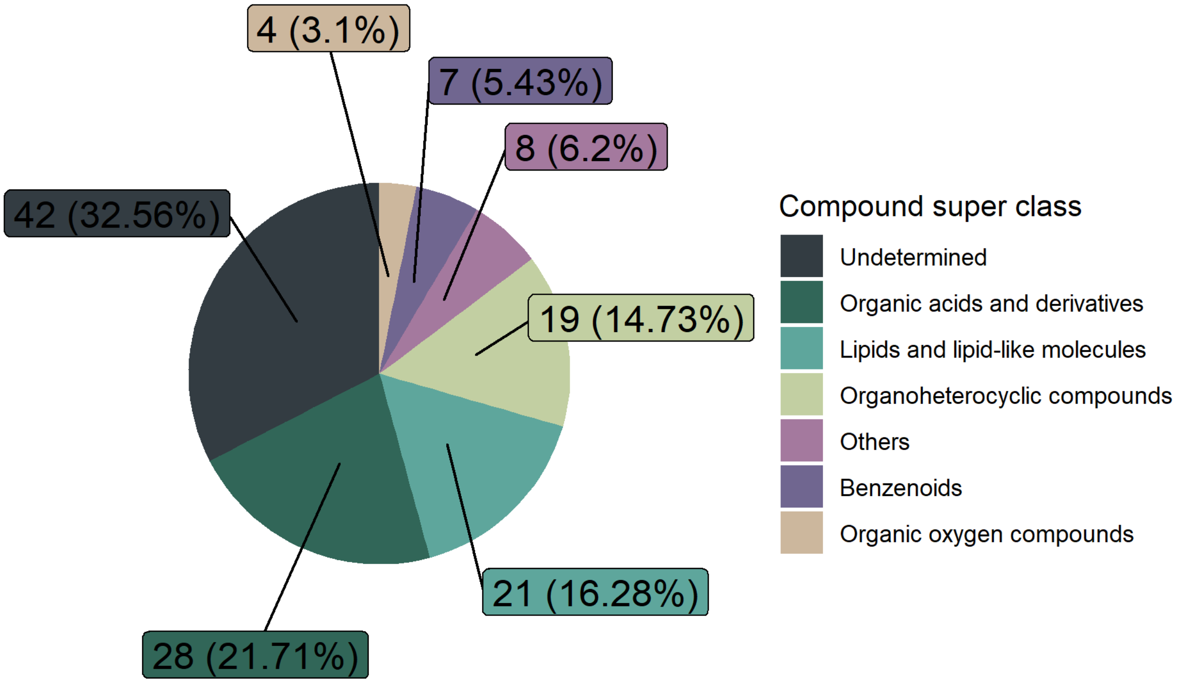

Fig. S2 Pie chart showing the chemical super class of annotated associated compounds (n = 129) identified in either of two analyses (using binary depression history or baseline GDS). Features annotated to multiple classes are categorized as undetermined in the plot.

Supplemental Figure 3

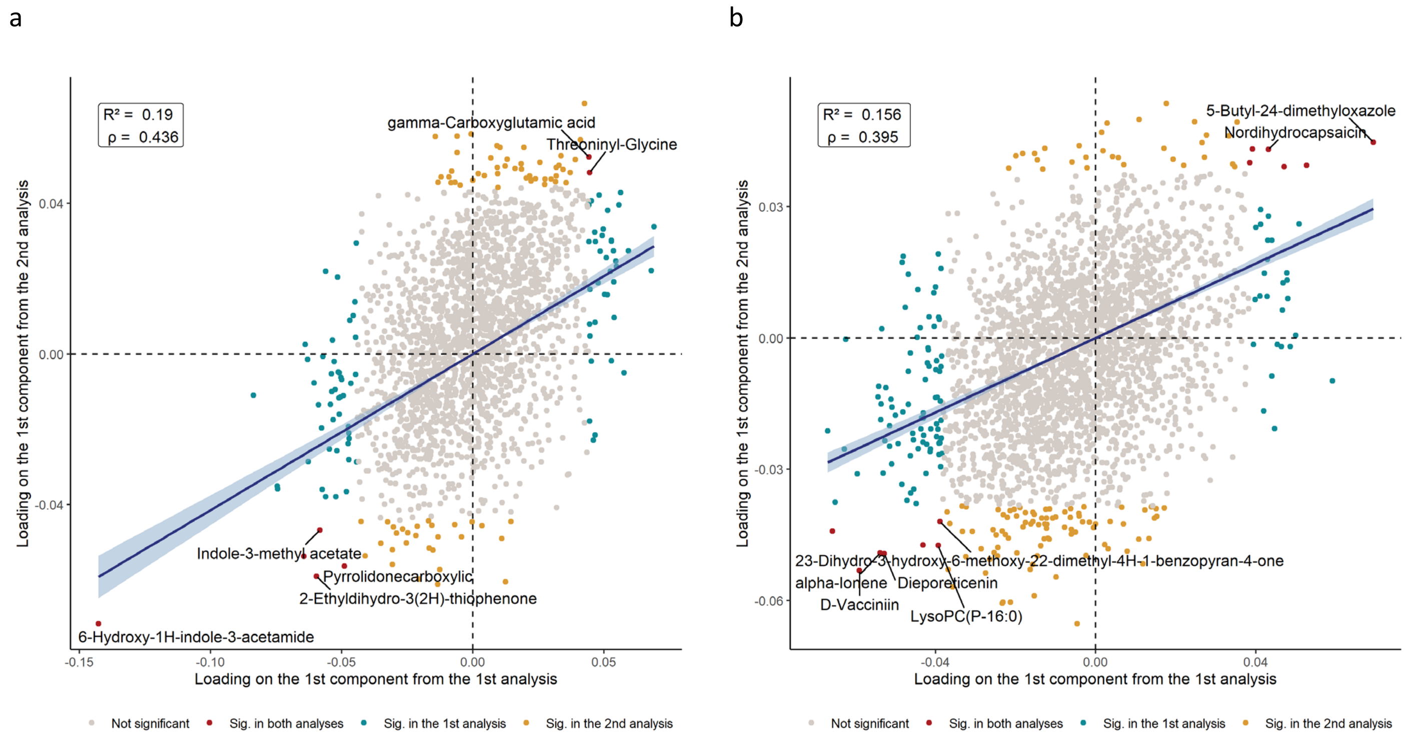

Fig. S3 Scatter plots showing the correlation between the estimated effect sizes from two MWAS. The X axis is the loading on the 1st component estimated from Analysis 1 (PLS-DA model using depression history as the outcome), and the Y axis is the loading on the 1st component estimated from Analysis 2 (PLS model using baseline GDS as the outcome). **Fig. S3a** shows the correlation pattern for the C18 (negative) column, and **Fig. S3b** shows the correlation pattern for the HILIC (positive) column. Annotated features with a VIP ≥ 2 in both analyses are labeled in the figure, with only the first annotation displayed when a feature maps to multiple compounds. The two MWAS using different depression outcomes showed moderate correlation and shared several overlapping features.

Supplemental Figure 4

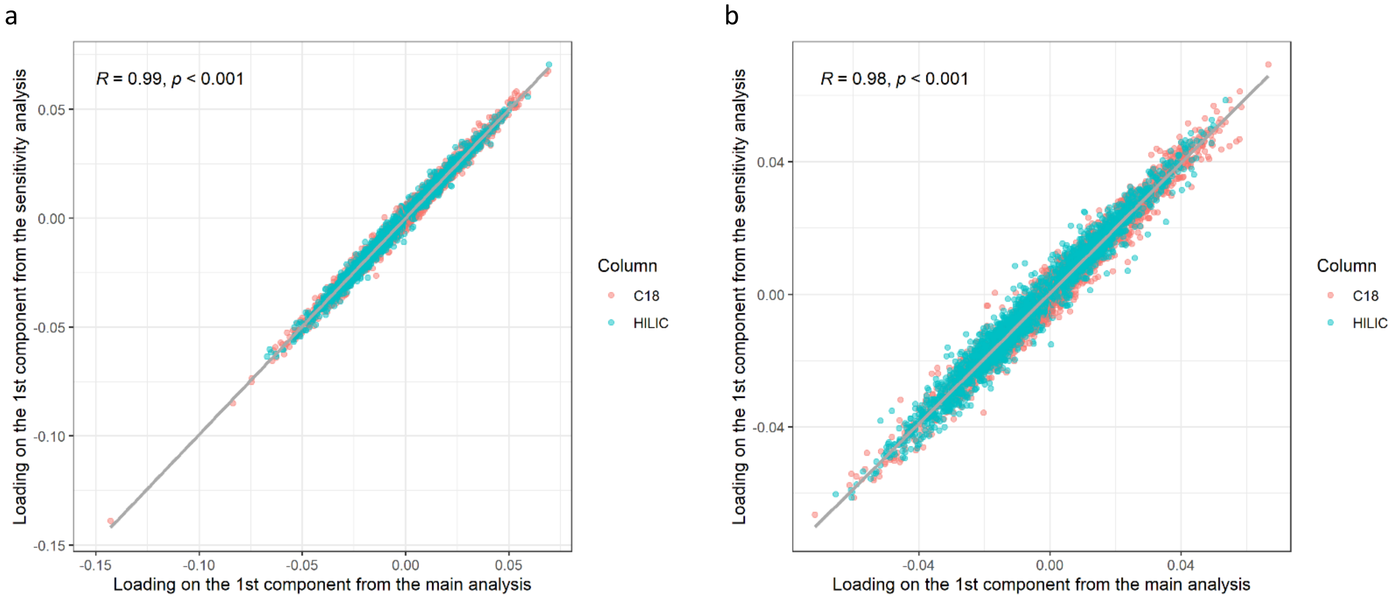

Fig. S4 Scatter plots showing the correlation between the estimated effect sizes from the main analysis and the sensitivity analysis with additional adjustment of LEDD. The X axis is the loading on the 1st component estimated from PLS-(DA) models in the main analysis, and the Y axis is the loading on the 1st component estimated in the sensitivity analysis that additionally adjusted for LEDD. **Fig. S4a** shows the correlation pattern for Analysis 1 using depression history as the outcome, and **Fig. S4b** shows the correlation pattern for Analysis 2 using GDS as the outcome. Pearson's correlation coefficients (R) between loadings from two analyses were calculated. The results before and after adjustment of LEDD are highly correlated. The inclusion of LEDD as an additional covariate overall did not change the results much.

Supplemental Figure 5

a B

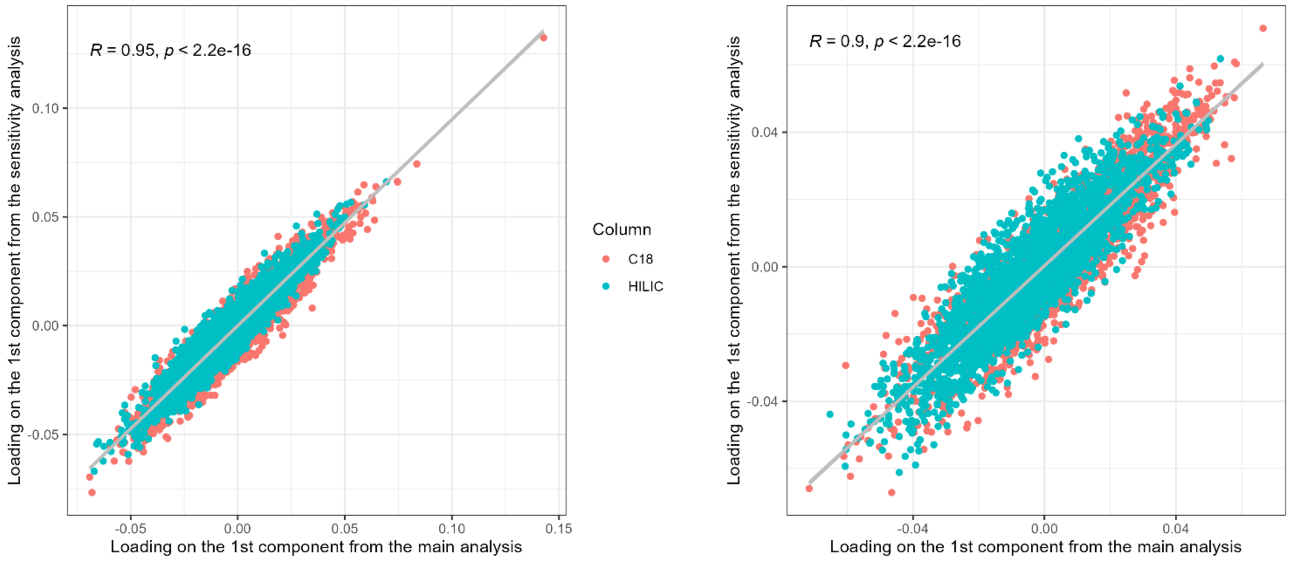

Fig. S5 Scatter plots showing the correlation between the estimated effect sizes from the main analysis and the sensitivity analysis with additional adjustment of the UPDRS-III, MMSE, and RBD indicator. The X axis is the loading on the 1st component estimated from PLS-(DA) models in the main analysis, and the Y axis is the loading on the 1st component estimated in the sensitivity analysis. **Fig. S5a** shows the correlation pattern for Analysis 1 using depression history as the outcome, and **Fig. S5b** shows the correlation pattern for Analysis 2 using GDS as the outcome. Pearson's correlation coefficients (R) between loadings from two analyses were calculated. The results before and after adjustment of extra PD-related clinical covariates are highly correlated. The inclusion of UPDRS-III, MMSE, and RBD indicator as additional covariates overall did not change the results much.

Supplemental Figure 6

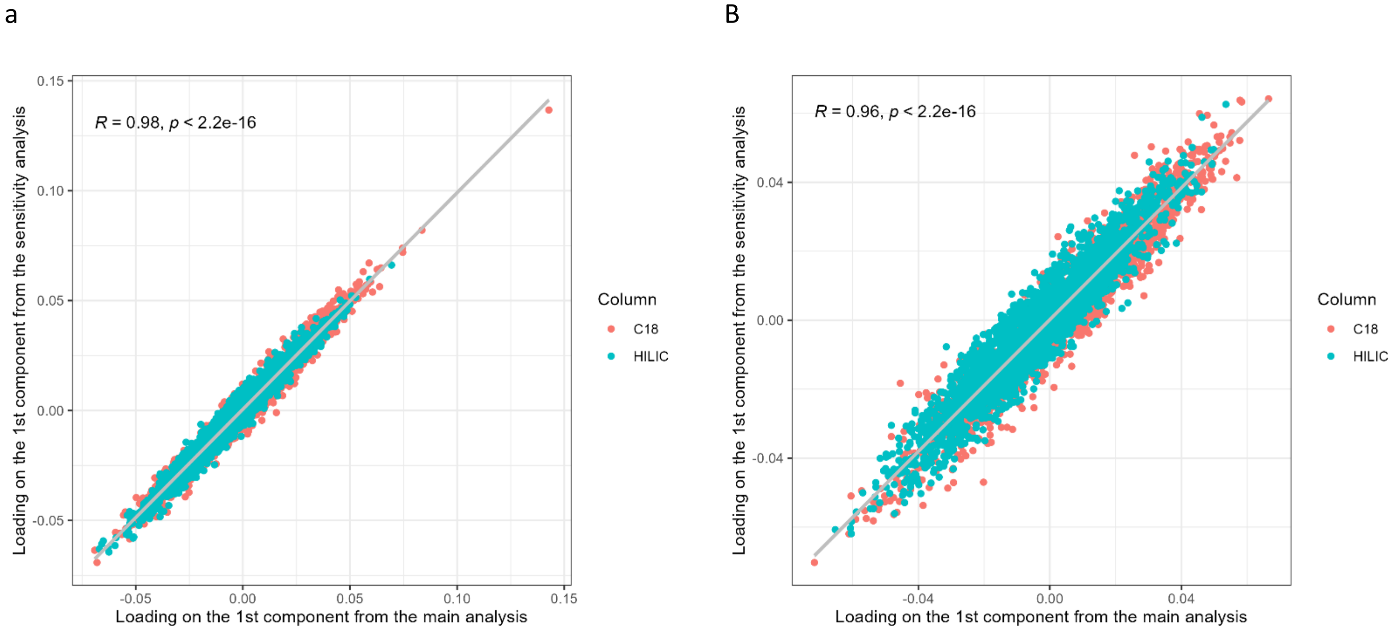

Fig. S6 Scatter plots showing the correlation between the estimated effect sizes from the main analysis and the sensitivity analysis with additional adjustment of the H&Y stage. The X axis is the loading on the 1st component estimated from PLS-(DA) models in the main analysis, and the Y axis is the loading on the 1st component estimated in the sensitivity analysis. **Fig. S6a** shows the correlation pattern for Analysis 1 using depression history as the outcome, and **Fig. S6b** shows the correlation pattern for Analysis 2 using GDS as the outcome. Pearson's correlation coefficients (R) between loadings from two analyses were calculated. The results before and after adjustment of H&Y stage are highly correlated. The inclusion of H&Y stage as additional covariates overall did not change the results much.

Supplemental Table 1. List of enriched pathways using "Mummichog" method in the sensitivity analysis additionally adjusted for LEDD.

	Pathway size	Total Number of peaks	Number of significant peaks	P value
**Analysis 1 (binary depression history)**				
Omega-3 fatty acid metabolism	39	10	6	0.000
Histidine metabolism	33	13	4	0.006
De novo fatty acid biosynthesis	106	26	10	0.007
Arginine and Proline Metabolism	45	19	4	0.014
Fatty acid activation	74	26	9	0.018
Glycerophospholipid metabolism	156	25	6	0.032
Linoleate metabolism	46	19	9	0.039
**Analysis 2 (baseline GDS score)**				
Glycerophospholipid metabolism	156	25	16	0.002
Biopterin metabolism	22	6	4	0.006
Tyrosine metabolism	160	65	27	0.009
Vitamin B9 (folate) metabolism	33	4	3	0.020
Sialic acid metabolism	107	18	13	0.023
Beta-Alanine metabolism	20	9	6	0.023
Pentose phosphate pathway	37	21	13	0.023
Caffeine metabolism	11	8	6	0.024
Tryptophan metabolism	94	34	15	0.028

Abbreviations: LEDD, levodopa-equivalent daily dosage; GDS, Geriatric Depression Scale.

Supplemental Table 2. List of enriched pathways using "Mummichog" method in the sensitivity analysis additionally adjusted for UPDRS-III, MMSE, and RBD indicator.

	Pathway size	Total Number of peaks	Number of significant peaks	P value*
**Analysis 1 (binary depression history)**				
Nitrogen metabolism	6	3	3	0.001
Glutamate metabolism	15	6	3	0.006
Alanine and Aspartate Metabolism	30	14	5	0.006
Arginine and Proline Metabolism	45	19	6	0.014
Vitamin B3 (nicotinate and nicotinamide) metabolism	28	9	3	0.014
TCA cycle	31	8	3	0.025
Omega-3 fatty acid metabolism	39	10	3	0.032
Methionine and cysteine metabolism	94	21	6	0.039
Valine, leucine and isoleucine degradation	65	14	4	0.062
Beta-Alanine metabolism	20	9	3	0.074
Purine metabolism	80	25	4	0.095
Glycerophospholipid metabolism	156	25	5	0.095
**Analysis 2 (baseline GDS score)**				
Glutamate metabolism	15	6	4	0.004
Caffeine metabolism	11	8	6	0.005
Glutathione Metabolism	19	6	4	0.005
Alanine and Aspartate Metabolism	30	14	8	0.013
Sialic acid metabolism	107	18	13	0.014
Galactose metabolism	41	22	16	0.014
Beta-Alanine metabolism	20	9	4	0.014
Pentose phosphate pathway	37	21	9	0.014
Glycine, serine, alanine and threonine metabolism	88	31	11	0.014
Tryptophan metabolism	94	34	12	0.017

Pyrimidine metabolism	70	22	9	0.038
Vitamin B9 (folate) metabolism	33	4	3	0.043
N-Glycan Degradation	16	5	4	0.043
Aspartate and asparagine metabolism	114	45	16	0.053
Arginine and Proline Metabolism	45	19	8	0.067
Chondroitin sulfate degradation	37	6	3	0.068
Biopterin metabolism	22	6	4	0.068
Heparan sulfate degradation	34	6	3	0.068
Pentose and Glucuronate Interconversions	15	11	7	0.068
Tyrosine metabolism	160	65	20	0.075

* Pathways with p-values less than 0.1 were displayed in this table, the pathways with p-values between 0.05 and 0.1 are colored in grey background.

Abbreviations: UPDRS-III, Unified Parkinson’s Disease Rating Scale Part III; MMSE, Mini Mental State Examination; RBD, of rapid eye movement sleep behavior disorder; GDS, Geriatric Depression Scale; TCA, tricarboxylic acid.

Supplemental Table 3. List of enriched pathways using "Mummichog" method in the sensitivity analysis additionally adjusted for H&Y stage

	Pathway size	Total Number of peaks	Number of significant peaks	P value
**Analysis 1 (binary depression history)**				
Omega-3 fatty acid metabolism	39	10	6	< 0.001
Nitrogen metabolism	6	3	2	0.009
De novo fatty acid biosynthesis	106	26	9	0.010
Glutathione Metabolism	19	6	3	0.021
Fatty acid activation	74	26	9	0.023
**Analysis 2 (baseline GDS score)**				
Tyrosine metabolism	160	65	26	0.002
Glycerophospholipid metabolism	156	25	13	0.005
Sialic acid metabolism	107	18	13	0.014
Pentose phosphate pathway	37	21	13	0.014
Vitamin B9 (folate) metabolism	33	4	3	0.014
Caffeine metabolism	11	8	6	0.016
Tryptophan metabolism	94	34	15	0.028
Biopterin metabolism	22	6	4	0.029

Abbreviations: H&Y stage, Hoehn and Yahr stage; GDS, Geriatric Depression Scale.

Supplemental Table 4. Depression medication names reported by patients and categories

Category	Cleaned medication names reported by patients
SSRI	Paroxetine, Sertraline, Fluoxetine, Citalopram, Escitalopram, Fluvoxamine, Sertraline HCL, Citalopram HBR, Vilazodone
SNRI	Venlafaxine, Duloxetine, Desvenlafaxine
Atypical Antidepressant	Bupropion, Trazodone, Nefazodone, Mirtazapine
Benzodiazepine	Alprazolam, Diazepam, Chlordiazepoxide, Lorazepam, Clonazepam
Others	Lithium, Imipramine, Meprobamate, Amitriptyline, Desipramine, Valproic Acid, Carbamazepine, Nortriptyline, Protriptyline, Propranolol, Lamotrigine, Metoprolol Succinate, Seroquel, Melatonin, Provigil, Gabapentin

Abbreviation: SSRI, Selective serotonin reuptake inhibitors; SNRI, Serotonin-Norepinephrine Reuptake Inhibitors.

Supplemental Table 5. Association between top peaks and use of different categories of depression medication

	Category	Unadjusted model		Adjusted Model 1 *		Adjusted Model 2 **	
		Beta (95% CI)	P value	Beta (95% CI)	P value	Beta (95% CI)	P value
**6-Hydroxy-1H-indole-3-acetamide**	**SSRI**						
	Never used	(ref)		(ref)		(ref)	
	Used more than 1 year ago	-1.36 (-2.26, -0.47)	**0.003**	-0.87 (-1.83, 0.08)	0.074	-0.89 (-1.85, 0.07)	0.070
	Using/used within 1 year	-3.62 (-4.20, -3.04)	**<0.001**	-3.07 (-3.77, -2.37)	**<0.001**	-3.12 (-3.82, -2.42)	**<0.001**
	**SNRI**						
	Never used	(ref)		(ref)		(ref)	
	Used more than 1 year ago	-0.92 (-3.39, 1.54)	0.462	0.46 (-1.92, 2.85)	0.702	0.47 (-1.91, 2.85)	0.697
	Using/used within 1 year	-3.62 (-4.87, -2.38)	**<0.001**	-2.33 (-3.56, -1.09)	**<0.001**	-2.32 (-3.56, -1.07)	**<0.001**
	**Atypical Antidepressant**						
	Never used	(ref)		(ref)		(ref)	
	Used more than 1 year ago	0.10 (-1.41, 1.62)	0.895	1.57 (0.11, 3.04)	**0.036**	1.65 (0.18, 3.12)	**0.028**
	Using/used within 1 year	-1.47 (-2.85, -0.09)	**0.037**	-0.23 (-1.56, 1.10)	0.734	-0.24 (-1.57, 1.08)	0.718
	**Benzodiazepine**						
	Never used	(ref)		(ref)		(ref)	
	Used more than 1 year ago	-2.36 (-4.66, -0.06)	**0.045**	-1.56 (-3.74, 0.62)	0.161	-1.70 (-3.88, 0.48)	0.127
	Using/used within 1 year	-0.03 (-2.33, 2.26)	0.977	0.42 (-1.76, 2.59)	0.707	0.46 (-1.71, 2.63)	0.675
	**Others**						
	Never used	(ref)		(ref)		(ref)	
	Used more than 1 year ago	-1.34 (-3.21, 0.53)	0.162	-0.08 (-1.87, 1.72)	0.934	-0.09 (-1.88, 1.71)	0.924
	Using/used within 1 year	-2.30 (-3.93, -0.67)	**0.006**	-1.67 (-3.21, -0.12)	**0.035**	-1.66 (-3.21, -0.12)	**0.035**
**L-Methionine**	**SSRI**						
	Never used	(ref)		(ref)		(ref)	
	Used more than 1 year ago	0.14 (0.00, 0.27)	**0.048**	0.11 (-0.06, 0.28)	0.192	0.11 (-0.05, 0.28)	0.190
	Using/used within 1 year	0.18 (-0.06, 0.42)	0.144	0.17 (-0.06, 0.41)	0.152	0.19 (-0.05, 0.43)	0.122
	**SNRI**						
	Never used	(ref)		(ref)		(ref)	
	Used more than 1 year ago	-0.02 (-0.28, 0.25)	0.911	-0.07 (-0.34, 0.21)	0.648	-0.08 (-0.36, 0.21)	0.600
	Using/used within 1 year	-0.34 (-0.93, 0.26)	0.265	-0.33 (-0.93, 0.26)	0.270	-0.34 (-0.93, 0.25)	0.260
	**Atypical Antidepressant**						
	Never used	(ref)		(ref)		(ref)	
	Used more than 1 year ago	-0.01 (-0.30, 0.28)	0.947	-0.06 (-0.35, 0.24)	0.716	-0.06 (-0.36, 0.24)	0.700
	Using/used within 1 year	-0.01 (-0.43, 0.42)	0.980	0.00 (-0.43, 0.43)	0.989	0.03 (-0.40, 0.46)	0.900
	**Benzodiazepine**						
	Never used	(ref)		(ref)		(ref)	
	Used more than 1 year ago	0.27 (-0.22, 0.75)	0.284	0.25 (-0.24, 0.74)	0.313	0.24 (-0.25, 0.73)	0.332
	Using/used within 1 year	0.20 (-0.48, 0.89)	0.562	0.21 (-0.47, 0.90)	0.539	0.19 (-0.50, 0.87)	0.596
	**Others**						
	Never used	(ref)		(ref)		(ref)	
	Used more than 1 year ago	-0.13 (-0.48, 0.22)	0.464	-0.15 (-0.50, 0.19)	0.390	-0.15 (-0.50, 0.19)	0.391
	Using/used within 1 year	-0.24 (-0.77, 0.28)	0.365	-0.22 (-0.74, 0.30)	0.412	-0.22 (-0.74, 0.30)	0.405

L-Tyrosine	**SSRI**						
	Never used	(ref)		(ref)		(ref)	
	Used more than 1 year ago	0.07 (-0.06, 0.19)	0.277	0.03 (-0.12, 0.18)	0.678	0.02 (-0.13, 0.17)	0.815
	Using/used within 1 year	0.01 (-0.20, 0.23)	0.903	0.01 (-0.21, 0.23)	0.933	0.02 (-0.20, 0.23)	0.891
	**SNRI**						
	Never used	(ref)		(ref)		(ref)	
	Used more than 1 year ago	0.06 (-0.19, 0.30)	0.653	0.01 (-0.24, 0.27)	0.934	0.02 (-0.23, 0.28)	0.872
	Using/used within 1 year	0.03 (-0.51, 0.57)	0.911	0.03 (-0.51, 0.58)	0.901	0.05 (-0.49, 0.59)	0.864
	**Atypical Antidepressant**						
	Never used	(ref)		(ref)		(ref)	
	Used more than 1 year ago	0.17 (-0.10, 0.44)	0.212	0.14 (-0.13, 0.41)	0.303	0.12 (-0.15, 0.40)	0.368
	Using/used within 1 year	0.00 (-0.39, 0.39)	0.982	0.00 (-0.39, 0.39)	0.998	0.02 (-0.37, 0.40)	0.937
	**Benzodiazepine**						
	Never used	(ref)		(ref)		(ref)	
	Used more than 1 year ago	0.16 (-0.28, 0.61)	0.467	0.15 (-0.29, 0.60)	0.505	0.15 (-0.29, 0.59)	0.504
	Using/used within 1 year	0.01 (-0.62, 0.63)	0.978	0.02 (-0.61, 0.64)	0.952	0.05 (-0.57, 0.67)	0.877
	**Others**						
	Never used	(ref)		(ref)		(ref)	
	Used more than 1 year ago	-0.15 (-0.46, 0.17)	0.357	-0.17 (-0.49, 0.15)	0.295	-0.16 (-0.48, 0.15)	0.307
	Using/used within 1 year	-0.27 (-0.75, 0.20)	0.264	-0.25 (-0.73, 0.23)	0.302	-0.25 (-0.72, 0.23)	0.303
(±)-2-Methylthiazolidine	**SSRI**						
	Never used	(ref)		(ref)		(ref)	
	Used more than 1 year ago	0.12 (-0.05, 0.28)	0.160	0.10 (-0.11, 0.30)	0.353	0.10 (-0.11, 0.30)	0.355
	Using/used within 1 year	0.13 (-0.16, 0.42)	0.369	0.13 (-0.16, 0.42)	0.380	0.14 (-0.15, 0.43)	0.338
	**SNRI**						
	Never used	(ref)		(ref)		(ref)	
	Used more than 1 year ago	0.02 (-0.30, 0.35)	0.895	-0.01 (-0.35, 0.32)	0.936	-0.02 (-0.36, 0.32)	0.905
	Using/used within 1 year	-0.55 (-1.27, 0.16)	0.130	-0.55 (-1.27, 0.17)	0.132	-0.56 (-1.27, 0.16)	0.130
	**Atypical Antidepressant**						
	Never used	(ref)		(ref)		(ref)	
	Used more than 1 year ago	0.01 (-0.34, 0.36)	0.956	-0.03 (-0.39, 0.33)	0.863	-0.04 (-0.40, 0.33)	0.848
	Using/used within 1 year	0.06 (-0.46, 0.58)	0.831	0.06 (-0.45, 0.58)	0.808	0.09 (-0.43, 0.61)	0.742
	**Benzodiazepine**						
	Never used	(ref)		(ref)		(ref)	
	Used more than 1 year ago	0.32 (-0.27, 0.90)	0.294	0.30 (-0.29, 0.89)	0.314	0.29 (-0.30, 0.88)	0.329
	Using/used within 1 year	0.06 (-0.77, 0.89)	0.892	0.07 (-0.76, 0.90)	0.874	0.04 (-0.79, 0.87)	0.920
	**Others**						
	Never used	(ref)		(ref)		(ref)	
	Used more than 1 year ago	-0.05 (-0.47, 0.37)	0.803	-0.07 (-0.49, 0.35)	0.745	-0.07 (-0.49, 0.35)	0.748
	Using/used within 1 year	-0.41 (-1.04, 0.22)	0.207	-0.39 (-1.03, 0.24)	0.226	-0.39 (-1.03, 0.24)	0.223

* Adjusted Model 1 adjusted for the history of depression diagnosis.

** Adjusted Model 2 adjusted for the history of depression diagnosis, race (non-Hispanic white or others), sex, and age

Abbreviation: SSRI, Selective serotonin reuptake inhibitors; SNRI, Serotonin-Norepinephrine Reuptake Inhibitors.

Supplemental Table 6. Association between top peaks and depression outcomes in PD controls

	Binary depression history*		Baseline GDS score*	
**Metabolite**	**Beta (95% CI)**	**p-value**	**Beta (95% CI)**	**p-value**
6-Hydroxy-1H-indole-3-acetamide	-0.16 (-0.31, 0.00)	**0.043**	-0.19 (-0.38, -0.01)	**0.041**
L-Methionine	0.34 (-0.21, 0.89)	0.228	-0.52 (-1.14, 0.09)	0.098
L-Tyrosine	0.14 (-0.47, 0.76)	0.651	-0.26 (-1.00, 0.48)	0.493
(±)-2-Methylthiazolidine	0.27 (-0.20, 0.74)	0.267	-0.01 (-0.45, 0.44)	0.973

* The transformed intensity of each metabolite was regressed on either binary depression history or baseline GDS score, adjusting for age at baseline interview, sex, education level, smoking history, and race/ethnicity.

Abbreviations: CI, confidence interval; GDS, Geriatric Depression Scale.

Supplemental Data

- **Supplemental Data 1.** Full metabolome-associated analysis results for Analysis 1 (using binary depression diagnosis as outcome).
- **Supplemental Data 2.** Full metabolome-associated analysis results for Analysis 2 (using baseline GDS score as outcome).
- **Supplemental Data 3.** Selected MWAS results from the sensitivity analysis compared to the main analysis (using depression history as the outcome).
- **Supplemental Data 4.** Selected MWAS results from the sensitivity analysis compared to the main analysis (using baseline GDS as the outcome).
- **Supplemental Data 5.** Selected MWAS results from the sensitivity analysis adjusted for UPDRS-III, MMSE, and RBD indicator, compared to the main analysis (using binary depression history as the outcome).
- **Supplemental Data 6.** Selected MWAS results from the sensitivity analysis adjusted for UPDRS-III, MMSE, and RBD indicator, compared to the main analysis (using baseline GDS as the outcome)
- **Supplemental Data 7.** Selected MWAS results from the sensitivity analysis adjusted for H&Y stage, compared to the main analysis (using binary depression history as the outcome)
- **Supplemental Data 8.** Selected MWAS results from the sensitivity analysis adjusted for H&Y stage, compared to the main analysis (using baseline GDS as the outcome)
- **Supplemental Data 9.** Selected MWAS results from the sensitivity analysis stratified by H&Y stage (early stage, H&Y <= 2)
- **Supplemental Data 10.** Selected MWAS results from the sensitivity analysis stratified by H&Y stage (late stage, H&Y >= 2.5)
